# Potential of desiccation-tolerant plant growth-promoting rhizobacteria in growth augmentation of wheat (*Triticum aestivum* L.) under drought stress

**DOI:** 10.3389/fmicb.2023.1017167

**Published:** 2023-02-08

**Authors:** Ajay Shankar, Vishal Prasad

**Affiliations:** Institute of Environment and Sustainable Development, Banaras Hindu University, Varanasi, India

**Keywords:** antioxidant, wheat, PGPR, drought, osmotic potential, antioxidative enzymes

## Abstract

Wheat (*Triticum aestivum* L.) yield and physiology are adversely affected due to limited water availability. However, desiccation-tolerant plant growth-promoting rhizobacteria (DT-PGPR) are potential candidates that can overcome the negative impacts of water stress. In the present study, a total of 164 rhizobacterial isolates were screened for desiccation tolerance up to −0.73 MPa osmotic pressure, of which five isolates exhibited growth and expression of plant growth properties under the influence of desiccation stress of −0.73 MPa. These five isolates were identified as *Enterobacter cloacae* BHUAS1, *Bacillus cereus* BHUAS2, *Bacillus megaterium* BHUIESDAS3, *Bacillus megaterium* BHUIESDAS4, and *Bacillus megaterium* BHUIESDAS5. All five isolates exhibited plant growth-promoting properties and production of exopolysaccharide (EPS) under the impact of desiccation stress. Furthermore, a pot experiment on wheat (variety HUW-234) inoculated with the isolates *Enterobacter cloacae* BHUAS1, *Bacillus cereus* BHUAS2, and *Bacillus megaterium* BHUIESDAS3 exhibited a positive influence on the growth of wheat under the condition of water stress. A significant improvement in plant height, root length, biomass, chlorophyll and carotenoid content, membrane stability index (MSI), leaf relative water content (RWC), total soluble sugar, total phenol, proline, and total soluble protein, were recorded under limited water-induced drought stress in treated plants as compared with non-treated plants. Moreover, plants treated with *Enterobacter cloacae* BHUAS1, *Bacillus cereus* BHUAS2, and *Bacillus megaterium* BHUIESDAS3 depicted improvement in enzymatic activities of several antioxidant enzymes such as guaiacol peroxidase (POD), catalase (CAT), and ascorbate peroxidase (APX). Beside this significant decrease in electrolyte leakage, H_2_O_2_ and malondialdehyde (MDA) contents were also recorded in treated plants. From the results obtained, it is evident that *E*. *cloacae* BHUAS1, *B*. *megaterium* BHUIESDAS3, and *B*. *cereus* BHUAS2 are the potential DT-PGPR having the capability to sustain growth and yield, alleviating the deleterious effect of water stress in wheat.

## 1. Introduction

Wheat is the second most important food grain after rice and provides 20% of the calories consumed by the world’s population, with a total annual production of ∼700 million tons worldwide (Qaseem et al., 2019). In rainfed areas, wheat cultivation faces heavy yield loss because of the irregular supply of water during the grain filling stage (Qaseem et al., 2019). Drought stress typically results in osmotic and oxidative stresses, which alter the physiological, biochemical, and molecular characteristics of plants and ultimately reduce crop yield. The decrease in plant growth and yield because of low water availability is essentially caused by altered plant water relations, reduced photosynthesis, oxidative stress at the cellular level, membrane degradation, and inhibited enzymatic activities. Any fluctuations in optimal values of physiological functions indicate the abnormality in plant health and their surrounding environmental conditions (Gangadhar et al., 2016; Batool et al., 2020). It is a serious challenge for breeders and practitioners to develop such a variety having genetic structure for high yield and resistance to abiotic stress for ensuring a sustainable agroecosystem under ever increasing environmental stresses (Lulsdorf et al., 2013; Batool et al., 2020). Genetic modification and transgenic methods have also been used for tolerance against drought in the case of many crops but their acceptance is under question due to social, ethical, and political issues (Jaiswal et al., 2022). Because of limited genetic diversity and ecological restrictions, further expansion in this way may be restricted for crop improvement (Xu and Zhou, 2006; Batool et al., 2020). On the other hand, crop management strategies to increase tolerance against adverse conditions can be a good option to overcome the impact and improve the yield under abiotic environmental stresses (Batool et al., 2020). In the past few years, plant-associated microorganisms such as plant growth-promoting rhizobacteria (PGPR) have gained attention for increasing crop productivity as well as their abiotic stress tolerance capabilities. The favorable impact in form of plant growth promotion has been reported in several crops (Saharan and Nehra, 2011; Adesemoye and Egamberdieva, 2013; Batool et al., 2020). The PGPR have been testified in improving seed germination, development of root and shoot, increasing biomass and chlorophyll content, soluble sugars and phenols, antioxidants, and water movement activity to maintain relative water content and uptake of nutrients in several studies (Meng et al., 2016; Sprenger et al., 2018; Batool et al., 2020). The PGPR represent a variety of root-colonizing bacterial species, which possess the ability to provide resistance to plants against several of the abiotic and biotic stressors owing to their root-colonizing capability (Mayak et al., 2004; Glick et al., 2007; Ngumbi and Kloepper, 2016). The mechanism behind the PGPR-mediated drought tolerance includes modification of root architecture, osmotic tolerance, managing oxidative stresses through biosynthesis of phytohormones and ACC-deaminase, reactive oxygen species (ROS) scavenging antioxidants, and production of exopolysaccharides (EPS), which help in biofilm formation for maintaining moisture availability in the root zone (Ngumbi and Kloepper, 2016; Vurukonda et al., 2016; Jochum et al., 2019). The soil having low water availability imposes desiccation stress on the resident microorganism. Bacteria that tolerate low water availability could be more viable and beneficial in such types of stressful soils; hence, the bacterial strains with higher tolerance toward desiccation stress perform better for plant growth promotion in such soils (Vilchez and Manzanera, 2011; Molina-Romero et al., 2017). Several bacteria such as *Rhizobium leguminosarum* (Casteriano et al., 2013), *Pseudomonas putida* mt-2 (Chang et al., 2007), *Bradyrhizobium japonicum* (Streeter, 2003), *Pseudomonas putida* KT2440 (van de Mortel et al., 2004), *Azospirillum brasilense* Sp7 (Molina-Romero et al., 2017), and *Pseudomonas putida* GAP-P45 (Sandhya et al., 2009) have been reported for their capabilities to tolerate desiccation stress as well as provide plant beneficial activities under drought stress in plants (Muñoz-Rojas, 2018). With this knowledge and background studies, the present study was carried out to isolate and characterize desiccation-tolerant plant growth-promoting bacteria and evaluate their efficacy in overcoming the negative impacts of low water-induced drought stress in wheat plants.

## 2. Materials and methods

### 2.1. Sampling site and physicochemical analysis of soil

A total of 24 rhizospheric soil samples were collected from roots of different crops grown in agricultural soils of the Hamirpur district of Uttar Pradesh, located between 25^°^27′00′′ to 25^°^57′00′′ N latitude and 79^°^11′00′′ to 80^°^19′00′′ E longitude with an area of 4139.09 km^2^ in the Bundelkhand plateau of the Ganga River Basin India, having various crops grown such as wheat, linseed, pigeon pea, mustard, pea, and gram. The soil physicochemical properties including soil moisture (SM), temperature (T), pH, water holding capacity (WHC), and soil texture along with soil nutrients such as organic carbon (OC), nitrogen (N), potassium (K), and phosphorus (P) were measured and analyzed using standard methodologies (Pawar and Shah, 2009).

### 2.2. Isolation and characterization of rhizobacteria

The isolation of rhizobacteria was performed by serial dilution using a plating technique on nutrient agar (NA) medium (Gouzou et al., 1993; Sandhya et al., 2009). The various bacterial isolates obtained were screened for desiccation tolerance in tryptone soya broth (TSB) medium having different water potentials (−0.05, −0.15, −0.30, −0.45, and −0.73 MPa) prepared using PEG-6,000 in an appropriate amount (Michel and Kaufmann, 1973; Sandhya et al., 2009). The desiccation-tolerant isolates obtained were further characterized based on their morphological characters such as shape, size, colony structure, gram staining, and biochemical properties (amylase, catalase, cellulase, citrate utilization, protease, and urease test) (Cappuccino and Sherman, 1992; Kammoun et al., 2008; Pereira and Castro, 2014). For molecular identification, bacterial genomic DNA of selected bacterial isolates was extracted using the method described by Sambrooke et al. (1989). The amplification of 16S rDNA genes was performed by polymerase chain reaction using universal primers 27F (5′-AGAGTTTGATCCTGGCTCAG-3′) and 1492R (5′-GTTACCTTGTTACGACTT-3′). The evolutionary genetics were studied using comparative molecular analysis, and the phylogenetic tree was constructed using MEGA-X (Kumar et al., 2004; Jaiswal et al., 2019).

### 2.3. Evaluation of plant growth-promoting (PGP) attributes and exopolysaccharide production of selected bacterial isolates

The PGP attributes of selected desiccation-tolerant bacterial isolates were carried out using established methods. Indole acetic acid (IAA) production was analyzed using the method of Bric et al. (1991), solubilization of insoluble phosphorus (P) was done by the method of Mehta and Nautiyal (2001), production of siderophore was analyzed using the method given by Schwyn and Neilands (1987), and production of ACC deaminase enzyme was estimated by calculating the quantity of α-ketobutyrate liberated along with ammonia after deamination of ACC was done by the method of Honma and Shimomura (1978) and Penrose and Glick (2003). All analyses were carried out in non-stress as well as desiccation stress (−0.73 MPa) under *in vitro* conditions. The estimation of protein content was done by the Bradford (1976) method. The exopolysaccharide (EPS) was estimated using a modification of the methods described by Bramhachari and Dubey (2006) and Kumar et al. (2011) under non-stress as well as under the condition of desiccation stress.

### 2.4. Application of selected bacterial isolates BHUAS1, BHUAS2, and BHUIESDAS3 on wheat

#### 2.4.1. Inoculum preparation and growing conditions

On the basis of the expression of PGP traits under the impacts of desiccation stress (−0.73 MPa), three bacterial isolates *Enterobacter cloacae* BHUAS1, *Bacillus megaterium* BHUIESDAS3, and *Bacillus cereus* BHUAS2 were selected for the evaluation of their growth-promoting effects on wheat plants grown under limited water condition. For the treatment of wheat seeds, the selected bacterial strains were grown in a 250 ml conical flask at 30 ± 2°C at 120 rpm for 24 h in a TSB medium. The bacterial cell density was maintained at 1 × 10^7^ cells/ml. For experiment work, wheat (*Triticum aestivum* L. var. HUW-234) seeds were procured from the seed bank of the Institute of Agricultural Sciences, Banaras Hindu University, Varanasi. Seeds were sterilized with 70% ethanol for 2 min followed by 0.1% HgCl_2_ for 3 min. In the next step, seeds were washed five times with sterile distilled water. After soaking in 1% CMC (adherent) for at least 10 h at room temperature, seeds were used for bacterial coating. Seeds were coated enough with bacterial cell suspension in double volume (1 × 10^7^ cells/ml) for 24 h. Three different treatments along with untreated control seeds were used — (1) **C:** Control (untreated) seeds, (2) **T1:** *Enterobacter cloacae* BHUAS1 coated seeds, (3) **T2:** *Bacillus megaterium* BHUIESDAS3 coated seeds, and (4) **T3:** *Bacillus cereus* BHUAS2 coated seeds.

The pot experiment was carried out at the Institute of Environment and Sustainable Development, Banaras Hindu University (25°15′44.17′′ N latitude and 82°59′41.59′′), Varanasi. The potting mixture used for the experiment contained 10 kg soil (9 kg of garden soil and 1 kg of farm yard manure) filled in a plastic pot of dimension 30cm × 20cm (height × width). The soil used in the experiment was from the garden of the Institute of Environment and Sustainable Development, Banaras Hindu University, Varanasi, India, mixed with farm yard manure and moistened before use. The final soil mix was found to have 12.9% sand, 36.9% clay, and 50.1% silt with water holding capacity of 41.2%. The pH and electrical conductivity (EC) of the soil were 6.94 μS/cm and 45.2 μS/cm, respectively. The organic carbon (OC), nitrogen (N), and available *P* in the soil were 0.6%, 52.71 kg/ha, and 49.5 kg/ha, respectively. The untreated and treated seeds were sown in plastic pots containing potting mixture. The pots were arranged in two sets: (1) Set I — non-stress (2) Set II — water stress on the basis of watering conditions. Each set has 12 pots with four types of treatment in triplicates, which have three bacterial treatments (T1: BHUAS1, T2: BHUIESDAS3, and T3: BHUAS2) and one control/untreated (C) pot. The seeds were allowed to germinate under environmental conditions and after germination thinning was performed by removing 50% of the seedling. At 10 DAS, 500 ml of sterilized tap water was added to each pot in both sets. At 20 DAS, watering was done again by adding 500 ml of sterilized tap water only in Set I (non-stress), while water limiting condition was induced by escaping watering in Set II (water stress). The total number of seeds germinated on each day was recorded. The percentage of seed germination was calculated on the basis of data recorded after 7 days. After 35 days, wheat plants were harvested, and different plant indices such as fresh weight, dry weight, root length, and shoot length were recorded using a standard protocol.

#### 2.4.2. Physiological analysis

##### 2.4.2.1. Electrolyte leakage

The electrolyte leakage (EL) was measured using the method described by Gonzalez and Gonzalez-Vilar (2003). Ten discs of wheat leaves were cut and placed in a test tube containing 10 ml of distilled water to estimate EL. The preliminary electrical conductivity (EC1) was measured. The tubes were kept at 10°C for 24 h before being placed in a 95°C water bath for 20 min. The samples were cooled, and the final electrical conductivity (EC2) was measured. The following formula was used to calculate the EL:


EL(%)=EC1EC2x 100

where EL represents electrolyte leakage and EC represents electrical conductivity.

##### 2.4.2.2. Membrane stability index

The membrane stability index (MSI) was calculated using a conductivity probe by following the modified method of Khanna-Chopra and Selote (2007). For this, a 1 cm piece of leaf was cut and washed with distilled water before being placed in test tubes with 10 ml of distilled water. The tubes were kept in a water bath at 40°C for 30 min. After that, tubes were taken out of the water bath and cooled down. A conductivity probe was used to measure the initial electrical conductivity (EC1). The samples were placed in a 100°C water bath for another 10 min. After cooling the samples, the final electrical conductivity (EC2) was measured again. The MSI was calculated using the formula as follows:


MSI=[1-(EC1EC2)]⁢x⁢ 100

where MSI is Membrane Stability Index and EC represents electrical conductivity.

##### 2.4.2.3. Relative water content

The relative water content (RWC) of leaves was determined using the standard method described by Teulat et al. (2003). For this, 1 g of fresh leaves were kept in 50 ml of distilled water in a 100 ml flask for 5 h at room temperature. The turgid weight was recorded, and the samples were oven dried for 2 h at 70°C to calculate the dry weight. The RWC was determined as follows:


RWC(%)=FW-DWTW-DWx 100

#### 2.4.3. Measurement of chlorophyll and carotenoid contents

Precisely weighed 0.5 g of the fresh leaf of wheat plant was placed in a test tube containing 10 ml of 80% acetone covered with a cap to stop evaporation of acetone and kept in dark overnight in a refrigerator at 4°C. The leaf sample along with acetone was then transferred to a mortar and pestle and homogenized. Furthermore, the homogenized sample was centrifuged at 10,000 rpm for 15 min at 4°C. The supernatant was transferred to a different tube, and then 0.5 ml of it was mixed with 4.5 ml of 80% acetone. This mixture was then analyzed for chlorophyll-a, chlorophyll-b, total chlorophyll, and carotenoids. The absorbance of the extracted sample was recorded by spectrophotometer (Thermo-Scientific, Evolution-201) at 645 and 663 nm for chlorophyll estimation and at 480 and 510 nm for carotenoids (Arnon, 1949; Sumanta et al., 2014). The calculation was done by using the following formula:


Chlorophyll⁢a⁢(mg/g)

$$=\frac{12.3\times \text{OD}\,\left(663\,n\,m\right)-0.85\times \text{OD}\,\left(645\,n\,m\right)}{\text{d}\times 1000\times \mathrm{weight}\,\left(g\right)}\,x\,V$$

Chlorophyll⁢b⁢(mg/g)

$$=\frac{19.3\times \text{OD}\,\left(645\,n\,m\right)-3.6\times \text{OD}\,\left(663\,n\,m\right)}{\text{d}\times 1000\times \text{weight}\,\left(\text{g}\right)}\,x\,V$$

Total⁢chlorophyll⁢(mg/g)

$$=\frac{20.2\times \text{OD}\,\left(645\,n\,m\right)-8.02\times \text{OD}\,\left(663\,n\,m\right)}{\text{d}\times 1000\times \mathrm{weight}\,\left(g\right)}\,x\,V$$

Carotenoids⁢(mg/g)

$$=\frac{7.6\times \text{OD}\,\left(480\,n\,m\right)-1.49\times \text{OD}\,\left(510\,n\,m\right)}{\text{d}\times 1000\times \mathrm{weight}\,\left(g\right)}\,x\,V$$

where

d = length of cuvette (1 cm), V = volume of extract (ml)

#### 2.4.4. Biochemical analysis

##### 2.4.4.1. Total soluble sugar

The method given by Dubois et al. (1956) was used to estimate the plant’s total soluble sugar using phenol sulphuric acid (PSA). For the total sugar analysis in the wheat plant, 0.1 g of fresh leaves were placed in 5 ml of 80% methanol and heated in a water bath for 1 h at 70°C. A 0.5 ml aliquot of this solution was taken and mixed with 0.5 ml of phenol (5%) and 1.5 ml of H_2_SO_4_ (96%). The solutions were thoroughly mixed and incubated in dark for 1 h at room temperature. After 1 h, the reaction mixture was thoroughly mixed by gentle shaking, and the absorbance at 490 nm was measured using a spectrophotometer (Thermo-Scientific, Evolution-201). The total sugar content was expressed as mg/g fresh tissue weight and calculated using the following formula:


Soluble⁢sugar⁢(mg/g)

$$=\frac{\mathrm{OD}\,\mathrm{of}\,\mathrm{sample}\,x\,K\,\mathrm{value}\,\left(20\right)}{\mathrm{weight}\,\mathrm{of}\,\mathrm{fresh}\,\mathrm{tissue}\,\left(0.5\,g\right)}\,x\,\mathrm{Dilution}\,\mathrm{factor}$$

##### 2.4.4.2. Total phenol

A total of 0.1 g of fresh leaf sample was homogenized in 2 ml of 80% methanol and heated for 15 min at 70°C to estimate total phenol (Zieslin and Ben-Zaken, 1993). After this, 1 ml of methanolic extract was mixed with 5 ml of distilled water and 250 μl of Folin–Ciocalteau reagent (1N). Following that, 1 ml of saturated sodium carbonate (20%) was added, and the mixture was incubated at 25°C for 30 min. A spectrophotometer was used to measure the absorbance at 725 nm (Thermo-Scientific, Evolution-201). The phenolic content was calculated using a Gallic acid standard curve and expressed as g GAE g^–1^ fresh weight.

##### 2.4.4.3. Proline and total soluble protein

Free proline in wheat plants was determined by the colorimetric method described by Bates et al. (1973). For this, 0.5 g of fresh leaf samples were homogenized with 10 ml of 3% aqueous sulfosalicylic acid, and the residue was removed by centrifugation at 12,000 rpm for 10 min at 4°C. From this, 1 ml of supernatant was taken and mixed with an equal volume of acid–ninhydrin and glacial acetic acid (1:1, v/v) in a test tube. The mixture was boiled for 1 h at 100°C. The reaction was terminated immediately by placing in ice water for 5 min and subsequently, 2 ml of toluene was added and vortexed for 2 min for the extraction of proline present in the mixture. The upper aqueous phase (wine red color) in toluene containing chromophores warmed at room temperature was collected, and its absorbance was recorded in a spectrophotometer (Thermo-Scientific, Evolution-201) at 520 nm. The proline concentration was calculated by using the L-proline standard curve and reported as μmol g^–1^ FW. The estimation of total protein concentration in wheat plants was determined according to the Bradford (1976) method.

##### 2.4.4.4. Reactive oxygen species, lipid peroxidation, and antioxidant enzymatic activities

Reactive oxygen species (ROS), lipid peroxidation (assessed by estimating malondialdehyde: MDA content), and antioxidant enzymatic activities were estimated in the leaf using the following methods. For the assay, 0.5 g of fresh leaves were cut and homogenized in a pre-chilled mortar and pestle with ice cold potassium phosphate (50 mM) extraction buffer (pH 7.0) and 0.4% (w/v) polyvinyl pyrrolidone (PVP). Furthermore, this homogenous mixture was centrifuged at 12,000 rpm for 30 min at 4°C. Then the supernatant was collected and used as a crude extract for the aforementioned assays using a UV-VIS spectrophotometer (Thermo-Scientific, Evolution-201).

The amount of H_2_O_2_ was measured using the method described by Alexieva et al. (2001). For this, the reaction mixture containing 0.5 ml of crude extract, 0.5 ml of 0.1 M potassium phosphate buffer, and 2 ml of KI (1 M) solution was incubated at room temperature in dark. The absorbance was recorded at 390 nm and 0.1% TCA was used as blank. The amount of H_2_O_2_ was calculated using a standard curve prepared using dilutions of a working standard of 100 μM of H_2_O_2_.

The MDA content was determined by a slight modification of the method described by Heath and Packer (1968) using thiobarbituric acid (TBA). For this, crude extract (0.3 ml) was mixed with 1.2 ml of 2-thiobarbituric acid (0.5% w/v) prepared in 20% trichloroacetic acid (TCA). The mixture was incubated at 95°C for 30 min. After that, the reaction was stopped by immediately immersing the tubes in an ice bath, and the mixture was centrifuged at 12,000 rpm for 10 min. The absorbance of the supernatant was measured using a UV-VIS spectrophotometer at 532 and 600 nm (Thermo-Scientific, Evolution-201). For non-specific absorbance, the absorbance at 600 nm was subtracted from the absorbance at 532 nm. The MDA concentration was determined using an extinction coefficient of 155 mM^–1^cm^–1^.


$$\mathrm{MDA}\,\mathrm{content}=\frac{\text{OD}\,\left(532\,n\,m\right)-\text{OD}\,\left(600\,n\,m\right)}{155}\,\mathrm{x1000}$$

Peroxidase (POD) activity was determined by adding 100 μL of enzyme extract in a reaction mixture (3.0 ml) containing 1.0 ml of 100 mM phosphate buffer (pH 7.0), 0.3 ml of 0.1 mM EDTA, 0.6 ml of 5.0 mM guaiacol, and 1.0 ml of 15 mM H_2_O_2_ described by Urbanek et al. (1991). The reaction started after the enzyme was added. In a UV-VIS spectrophotometer, the absorbance increased for 90 s at 470 nm (Thermo-Scientific, Evolution-201). The amount of tetraguaiacol formed was quantified using its molar extinction coefficient (26.6 mM^–1^cm^–1^), and enzyme activity was expressed as mol min^–1^ mg^–1^ protein.

For CAT activity, 200 μl of enzyme extract was added to the reaction mixture (3.0 ml) containing 1.5 ml of 50 mM phosphate buffer (pH 7.0), 300 μl of 0.1 M H_2_O_2_, and 1.0 ml of distilled water. The CAT activity was assayed using a spectrophotometer by monitoring the decrease in the absorbance of H_2_O_2_ at 240 nm (Bin et al., 2010; Batool et al., 2020). The reaction was started by adding an aliquot of the enzyme to the reaction mixture. The absorbance change was monitored 90 s after the reaction began. The absorbance difference (A240) was divided by the H_2_O_2_ molar extinction coefficient (39.4 M^–1^cm^–1^). The enzyme activity was expressed in mol min^–1^ mg^–1^ protein.

The activity of APX was measured using a slightly modified method developed by Nakano and Asada (1981). 200 μl of enzyme extract was added to the reaction mixture of 50 mM sodium phosphate buffer (pH 7.0), 0.2 mM EDTA, and 0.5 mM ascorbic acid. H_2_O_2_ was added to a final concentration of 0.1 mM to start the reaction. The oxidation of ascorbic acid was detected as a decrease in the absorbance at 290 nm using a UV-VIS spectrophotometer (Thermo-Scientific, Evolution-201) 90 s after the reaction began. The difference in absorbance was divided by the ascorbate molar extinction coefficient (2.8 mM^–1^cm^–1^). The enzyme activity was expressed as nmol of H_2_O_2_ min^–1^mg^–1^ protein taking into consideration that 1.0 mol of ascorbate is required for the reduction of 1.0 mol of H_2_O_2_.

### 2.5. Statistical analysis

All the data obtained were considered for correlation analysis (5% significance levels) and illustrated as a graph. The results were expressed as mean ± SD of three independent replicates. Analysis of variance (ANOVA) was done followed by Duncan’s multiple range test (DMRT) to compare the means and determine the significant differences between each treatment. The level of statistical significance was set to *P* < 0.05.

## 3. Results

### 3.1. Soil characteristics, isolation, and screening of desiccation-tolerant rhizobacteria

**FIGURE 1:**
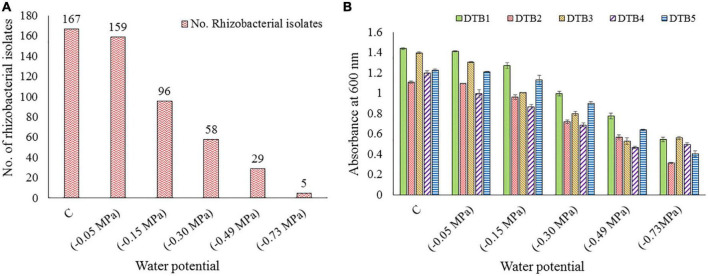
**(A)** Total number of different bacterial isolates obtained from various soil samples and further surviving isolate count at various levels of desiccation stress imposed by PEG-6000, **(B)** growth performance of five selected desiccation-tolerant bacterial isolates at various levels of desiccation stress imposed by PEG-6000.

The pH of the soil samples varied from slightly acidic (6.35) to alkaline (9.13). On average, soil comprised 41.38% clay, 36.86% silt, and 21.76% sand content. The average SM was observed to be 21.5%. The average value for temperature, EC, and WHC was 17.9°C, 68.8 μs/cm, and 45.4%, respectively. The nutrients N, P, and K were estimated to be 30.32, 133.04, and 284.01 kg/ha, respectively, whereas the OC content was 1.36%. A total of 167 rhizobacteria were isolated, which were further screened for desiccation tolerance. Only five isolates were observed to survive and grow at maximum desiccation stress of 0.73 MPa (Figure 1A). They were named as DTB1, DTB2, DTB3, DTB4, and DTB5. Among them, isolate DTB3 showed maximum growth at −0.73 MPa water potential. The overall growth behavior of all five isolates was observed to be in the following order DTB3 > DTB1 > DTB4 > DTB5 > DTB2 (Figure 1B) at −0.73 MPa.

### 3.2. Morphological, biochemical, and molecular characterization of bacterial isolates

All the isolates were rod shaped among which four were Gram positive, whereas one isolate was Gram negative (Table 1). Isolate DTB1 exhibited positive activity for catalase, citrate, and urease. Isolate DTB2 was positive for catalase, cellulase, and urease. Isolate DTB3 was positive for catalase, cellulase, urease, and protease. Isolate DTB4 showed positive activity for amylase, catalase, cellulase, citrate, urease, and protease, while isolate DTB5 was positive for amylase, catalase, cellulase, urease, and protease (Table 1).

**TABLE 1:** Morphological and biochemical characteristics of five selected desiccation-tolerant rhizobacterial isolates.

Characteristics		Desiccation-tolerant rhizobacterial isolates				
		DTB1	DTB2	DTB3	DTB4	DTB5
**Morphological**						
Colony morphology-	Form	Circular	Circular	Circular	Circular	Irregular
	Elevation	Convex	Crateriform	Crateriform	Raised	Flat
	Margin	Entire	Entire	Entire	Undulate	Undulate
	Color	Off white	Cream Yellow	Cream Yellow	Cream Yellow	White
	Appearance	Slimy	Slimy	Slimy	Slimy	Slightly Slimy
Cell morphology-	Gram’s test	Negative	Positive	Positive	Positive	Positive
	Shape	Rod	Rod	Rod	Rod	Rod
**Biochemical**						
Amylase		–	–	–	+	+
Catalase		+	+	+	+	+
Cellulase		–	+	+	+	+
Citrate		+	–	–	+	–
Protease		–	–	+	+	+
Urease		+	+	+	+	+

**FIGURE 2:**
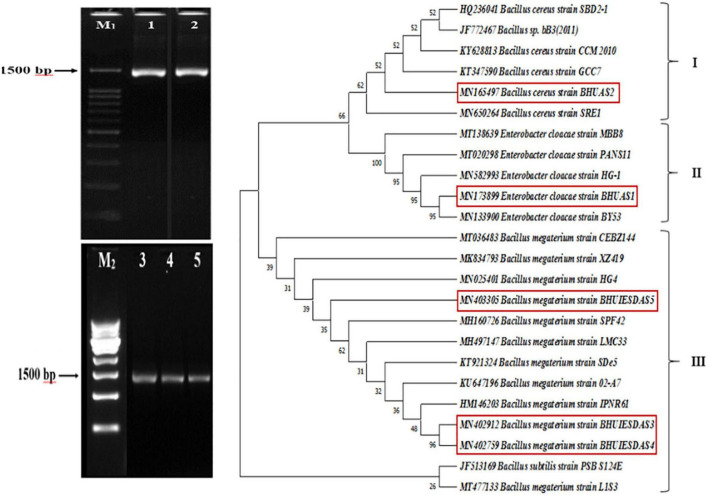
Gel-electrophoresis image showing the 1,500 bp amplified product of 16S rDNA gene from the five selected desiccation-tolerant rhizobacterial isolates 1-DTB1, 2-DTB5, 3-DTB2, 4-DTB3, and 5-DTB4. M1 is a 100 bp ladder and M2 is a 500 bp ladder. Phylogenetic tree obtained by the UPGMA method and bootstrap clustering showing the similarity relatedness of the selected rhizobacterial isolates and their relative position in respective clusters.

Blast similarity analysis revealed that bacterial isolate DTB1 exhibited 92% similarity with *Enterobacter cloacae* strain PANS11, DTB2 showed 96% similarity with *Bacillus megaterium* strain IPNR61, DTB3 had 99% similarity with *B. megaterium strain 02-A7*, DTB4 showed 100% similarity with *B. megaterium* strain CEBZ144, and DTB5 showed 99.71% similarity with *B. cereus* strain SRE1. The sequences were deposited in the NCBI Gene-Bank database and assigned accession numbers MN173899, MN402912, MN402759, MN403305, and MN165497 were obtained. The phylogenetic analysis revealed three different clusters (Figure 2) of which cluster III had a majority of assemblage (14) consisting of three isolates of *B. megaterium* BHUIESDAS3 (DTB2), *B. megaterium* BHUIESDAS4 (DTB3), and *B. megaterium* BHUIESDAS5 (DTB4). *B. cereus* BHUAS2 (DTB5) was clustered with five other bacterial species in cluster I. Cluster II had *E. cloacae* BHUAS1 (DTB1) with five reference bacterial species.

### 3.3. Estimation of IAA, P-solubilisation, siderophore, ACCD, and EPS

**FIGURE 3:**
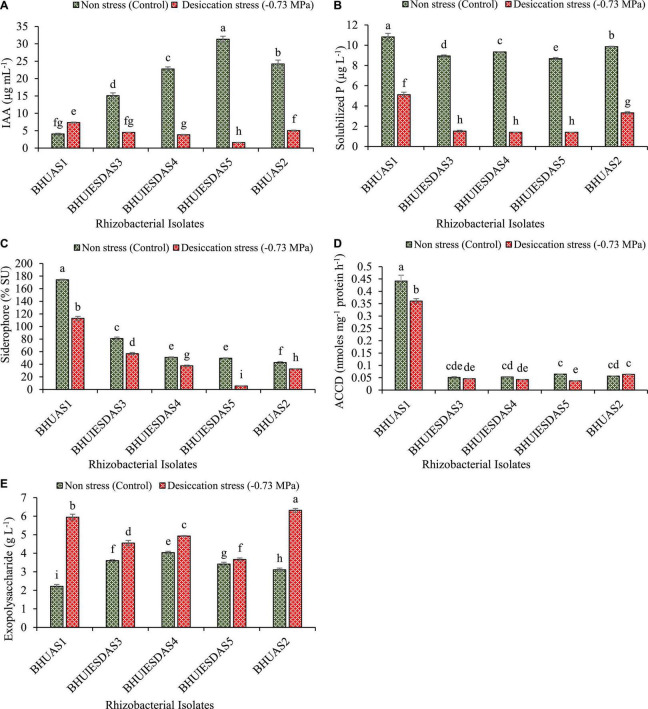
Expression of plant growth-promoting features of five selected desiccation-tolerant bacterial strains under non-stress and impacts of desiccation stress **(A)** amount of IAA produced, **(B)** amount of solubilized phosphate liberated, **(C)** amount of Fe chelating siderophore produced, **(D)** amount of ACC deaminase expressed, and **(E)** amount of exopolysaccharide produced. The different alphabets indicate statistically significant differences between each treatment (DMRT *p <* 0.05).

The strains *Enterobacter cloacae* BHUAS1, *Bacillus megaterium* BHUIESDAS3, *Bacillus megaterium* BHUIESDAS4, *Bacillus megaterium* BHUIESDAS5, and *Bacillus cereus* BHUAS2 exhibited production of IAA, solubilization of insoluble phosphate, production of siderophore, and ACC deaminase activity under desiccation stress (−0.73 MPa water potential) (Figure 3). Expression of all PGP properties was observed under the impacts of desiccant by all the strains with a significant increase in EPS production under desiccation stress. *Enterobacter cloacae* BHUAS1 was observed to produce the highest amount of IAA under desiccation stress followed by *Bacillus cereus* BHUAS2, *Bacillus megaterium* BHUIESDAS3, *Bacillus megaterium* BHUIESDAS4, and *Bacillus megaterium* BHUIESDAS5 (Figure 3A). The amount of soluble phosphate was found significantly high in *Enterobacter cloacae* BHUAS1 under stress as well as desiccation challenge in comparison to others (Figure 3B). Furthermore, the production of siderophore was observed to be significantly high again in the case of *Enterobacter cloacae* BHUAS1 under no stress as well as desiccation stress in comparison to others (Figure 3C). *Enterobacter cloacae* BHUAS1 was also observed to produce the highest amount of ACC deaminase both under control as well as desiccation stress (Figure 3D). The EPS production was highest in *Bacillus cereus* BHUAS2 (6.31 g L^–1^), followed by *Enterobacter cloacae* BHUAS1 (5.95 g L^–1^), *Bacillus megaterium* BHUIESDAS4 (4.93 g L^–1^), *Bacillus megaterium* BHUIESDAS3 (4.55 g L^–1^), and *Bacillus megaterium* BHUIESDAS5 (3.66 g L^–1^) (Figure 3E).

### 3.4. Growth improvement in wheat under limited water-induced drought stress

**FIGURE 4:**
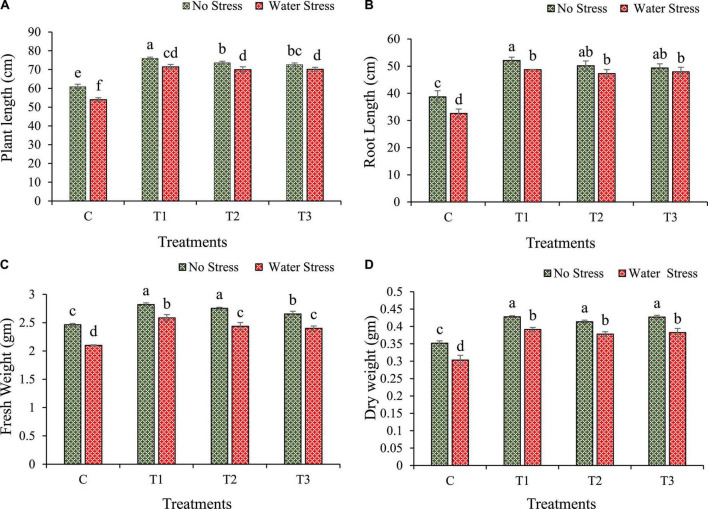
Impacts of bacterial treatments (T1 — *Enterobacter cloacae* BHUAS1, T2–*Bacillus megaterium* BHUIESDAS3, and T3 — *Bacillus cereus* BHUAS2) on various morpho-physiological features of wheat plants under the influence of water-induced drought stress **(A)** plant height, **(B)** root length, **(C)** fresh weight, and **(D)** dry weight. Data are presented as means ± SD of three replicates. The different alphabets indicate statistically significant differences between each treatment (DMRT *p <* 0.05).

Bacterial treatment was observed to have a substantial effect on seed germination as compared with non-treated seeds. Highest seed germination was observed with *Enterobacter cloacae* BHUAS1 (T1) (88.89%) followed by *Bacillus cereus* BHUAS2 (T3) (88.78%) and *Bacillus megaterium* BHUIESDAS3 (T2) (85.56%), while control showed 77.78% germination of total seed sown after 7 days. The stress imposed by limited water availability led to a significant reduction in the growth dynamics of the plant (Figure 4). Plants with bacterial treatment showed better performance under drought than plants without bacterial treatment. It was observed that during the condition of water stress, plant height was increased by 32, 29, and 30% in T1, T2, and T3 inoculated plants, respectively, in comparison to un-inoculated control plants (Figure 4A). Root length was increased by 49, 45, and 47% in T1, T2, and T3 inoculated plants, respectively, than plants without inoculation (control) under water stress (Figure 4B). Similarly, under water stress, fresh weight of plant was enhanced by 23, 16, and 14% in case of T1, T2, and T3 treated plants, respectively, than in untreated control plants (Figure 4C), while dry weight was improved by 29, 24, and 26% in plants treated with T1, T2, and T3, respectively, as compared with un-inoculated control plant (Figure 4D).

### 3.5. Changes in leaf chlorophyll and carotenoid content

**FIGURE 5:**
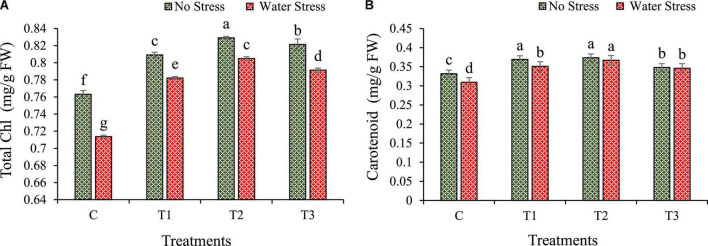
Impacts of bacterial treatments (T1 — *Enterobacter cloacae* BHUAS1, T2 — *Bacillus megaterium* BHUIESDAS3, and T3 — *Bacillus cereus* BHUAS2) on various biochemical contents of wheat plants under the influence of water-induced drought stress **(A)** total chlorophyll and **(B)** carotenoid. The different alphabets indicate statistically significant differences between each treatment (DMRT *p <* 0.05).

The plant pigments such as total chlorophyll and carotenoid of wheat plants were increased in bacterial treated plants under the influence of water stress (Figure 5). During water stress, total chlorophyll was enhanced by 10, 13, and 11% in plants with T1, T2, and T3 treatment, respectively, as compared to plants without bacterial inoculation. The T1 treated plants showed maximum chlorophyll content (Figure 5A). Similarly, under water stress, carotenoid content was also improved in wheat plants with bacterial treatment over the untreated control plants. During water stress conditions, carotenoid content increased by 13, 19, and 12% in T1, T2, and T3 treated plants, respectively, than plants without bacterial treatment (Figure 5B).

### 3.6. Changes in plant physiological parameters

**FIGURE 6:**
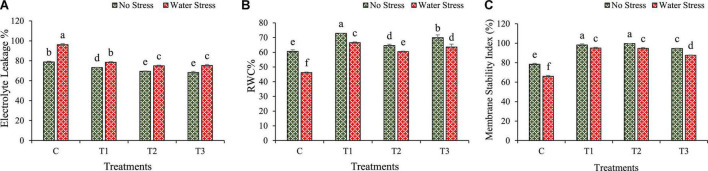
Impacts of bacterial treatments (T1 — *Enterobacter cloacae* BHUAS1, T2 — *Bacillus megaterium* BHUIESDAS3, and T3 — *Bacillus cereus* BHUAS2) on various features of wheat plants under the influence of water-induced drought stress **(A)** electrolyte leakage (EL), **(B)** relative water content (RWC), and **(C)** membrane stability index (MSI). The different alphabets indicate statistically significant differences between each treatment (DMRT *p <* 0.05).

Changes in physiological traits such as EL, RWC, and MSI are calculated under water stress in wheat plants as shown in Figure 6.

The water stress resulted in an increased level of electrolyte leakage (EL) in wheat plants (Figure 6A). However, under water stress, plants having bacterial inoculation showed a decrease in EL than in plants without bacterial treatment. During water stress, EL decreased by 18, 22, and 21% in T1, T2, and T3 treated wheat plants, respectively, as compared with un-inoculated plants.

The water stress was also observed to decrease the relative water content (RWC) and membrane stability index (MSI). However, under water stress, wheat plants with bacterial treatments exhibited an increment in RWC than plants without bacterial treatment (Figure 6B). Under stress conditions, RWC was enhanced by 44, 31, and 38% in plants inoculated with T1, T2, and T3, respectively, in comparison to plants without treatment.

The membrane stability decreased under water stress in wheat plants. However, under water stress, the plants with bacterial treatments showed an increase in MSI than plants without bacterial inoculation (Figure 6C). During water stress, MSI was improved by 44, 43, and 33% in plants with T1, T2, and T3 bacterial treatments, respectively, than in plants without bacterial treatment.

### 3.7. Changes in lipid peroxidation and reactive oxygen species

**FIGURE 7:**
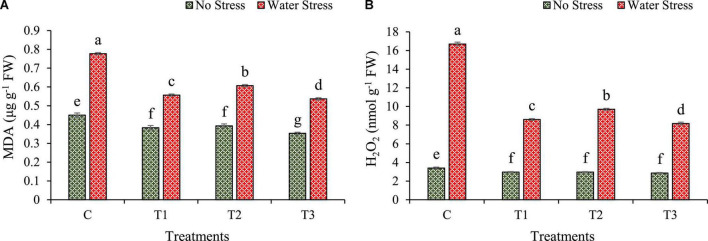
Impacts of bacterial treatments (T1 — *Enterobacter cloacae* BHUAS1, T2 — *Bacillus megaterium* BHUIESDAS3, and T3 — *Bacillus cereus* BHUAS2) on various features of wheat plants under the influence of water-induced drought stress **(A)** malondialdehyde (MDA) and **(B)** hydrogen peroxide (H_2_O_2_). The different alphabets indicate statistically significant differences between each treatment (DMRT *p <* 0.05).

Changes in malondialdehyde (MDA) and reactive oxygen species (ROS) such as hydrogen peroxide (H_2_O_2_) under water stress are calculated in wheat plants as shown in Figure 7.

The water stress led to an increase in lipid peroxidation quantitated by the synthesis of MDA (Figure 7A). However, bacterial treated plants depicted a lower production of MDA in comparison to plants without bacterial inoculation under both no stress as well as water stress. The MDA content was decreased by 28, 22, and 31% in the case of plants treated with T1, T2, and T3, respectively, as compared to plants without any treatment under water stress.

Due to water stress, H_2_O_2_ production was increased in wheat plants (Figure 7B). However, plants having bacterial treatments showed a lower amount of H_2_O_2_ produced under water stress than plants without bacterial treatments. In wheat plants under water stress, H_2_O_2_ production decreased by 49%, 42%, and 51% in bacterial treatments T1, T2, and T3, respectively, in comparison to plants without treatments.

### 3.8. Changes in antioxidant enzyme activities

**FIGURE 8:**
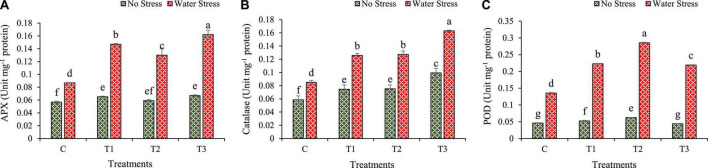
Impacts of bacterial treatments (T1 — *Enterobacter cloacae* BHUAS1, T2 — *Bacillus megaterium* BHUIESDAS3, and T3 — *Bacillus cereus* BHUAS2) on activities of various antioxidant enzymes of wheat plants under the influence of water-induced drought stress **(A)** ascorbate peroxidase (APX), **(B)** catalase (CAT), and **(C)** peroxidase (POD). The different alphabets indicate statistically significant differences between each treatment (DMRT *p <* 0.05).

Water stress mostly activates the production of antioxidant enzymes and modulates plant physiology. It was found that during water stress, the production of ascorbate peroxidase (APX), catalase (CAT), and peroxidase (POD) increased in inoculated plants more than in un-inoculated plants (Figure 8).

The activity of APX was increased under water stress in wheat plants and was found higher than in no stress conditions (Figure 8A). However, plants with bacterial treatments T1, T2, and T3 showed an increase in APX activity by 70, 50, and 87%, respectively, in comparison to plants without bacterial treatment under water stress conditions.

Under water stress, the CAT activity increased. It was found higher under water stress as compared with no stress. During water stress condition, the plants having bacterial treatments depicted higher CAT activity as compared with un-inoculated plants (Figure 8B). The CAT activity was increased by 48, 50, and 92%, respectively in plants treated with T1, T2, and T3 over control plants under water stress.

Similarly, the POD activity also increased significantly under water stress. The plants with bacterial treatments showed higher POD activity as compared to plants without bacterial treatments under water stress condition (Figure 8C). The POD activity increased by 64, 111, and 62%, respectively, in plants inoculated with T1, T2, and T3 as compared to plants without bacterial treatment under water stress.

### 3.9. Changes in the production of biochemicals

**FIGURE 9:**
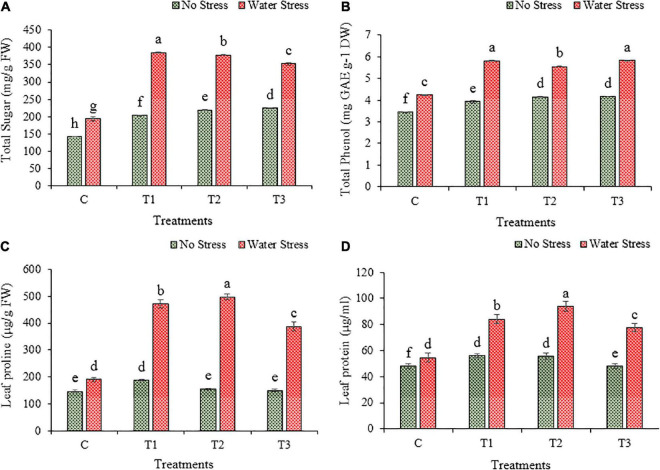
Impacts of bacterial treatments (T1- *Enterobacter cloacae* BHUAS1) (T2- *Bacillus megaterium* BHUIESDAS3) (*Bacillus cereus* BHUAS2) on various features of wheat plants under the influence of water induced drought stress **(A)** total sugar, **(B)** total phenol, **(C)** proline content, and **(D)** protein content. The different alphabets indicate statistically significant differences between each treatment (DMRT *p* < 0.05).

The production of soluble sugar was increased under the impact of water stress than no stress in wheat plants (Figure 9A). During water stress, the plants inoculated with bacterial isolates showed higher production than plants without inoculation. The production of total soluble sugar was increased by 99, 95, and 82% in plants treated with bacteria T1, T2, and T3, respectively, than in un-inoculated plants under water stress.

The content of phenol increased due to water stress in wheat plants (Figure 9B). Under water stress conditions, the plants with bacterial treatments showed higher phenol content as compared to plants without bacterial inoculation. The phenol content was increased by 37, 31, and 38% in wheat plants with inoculation of T1, T2, and T3, respectively, in comparison to plants without any treatment under water stress.

Due to water stress, the proline content was increased significantly in wheat plants (Figure 9C). More production of proline was observed in plants having inoculation with bacterial isolates than in un-inoculated plants. The proline content was increased by 148, 161, and 104% in T1, T2, and T3 treated plants, respectively, than plants without bacterial treatment under water stress.

Similarly, water stress resulted in an increased production of total soluble protein in wheat plants (Figure 9D). Under water stress, the plants inoculated with bacterial isolates depicted higher production of soluble protein than plants without bacterial treatment. The production of total soluble protein increased by 54, 72, and 42% in plants with the application of T1, T2, and T3, respectively, than in plants without bacterial application under water stress.

## 4. Discussion

Drought is one of the most disastrous abiotic stressors in agriculture, with particular importance in wheat cultivars because of their vulnerability to low water which results in a significantly decreased physiological growth and yield under the impact of drought (Gaju et al., 2009; Qaseem et al., 2019). The history of the use of plant beneficial microorganisms such as PGPR in agriculture is too old, and it has been increasing day by day in enhancing plant’s tolerance to different adverse environmental stresses including drought (Lizana et al., 2006; Batool et al., 2020). The establishment of the association of soil with plant roots created a rhizosphere system in which plants secreted exudates for inhabiting microflora so that they could flourish with the food and other nutrients such as C, N, and *P* (Jaiswal et al., 2022). The bacteria-associated roots of the plant play a crucial role in plant growth and development through different mechanisms (Chouhan et al., 2021; Jaiswal et al., 2022). Most often bacteria present in soil face the condition of desiccation due to water deficit, which leads to the development of a natural tolerance in them for growth and survival under water deficit conditions (Potts, 1994; Alpert, 2005). In the present study, the count of bacterial isolates obtained differently from soil samples was 167, which displayed a significant variation in their physicochemical properties. The variations in parameters such as WHC, SOC, *P*, and K are known to have a major impact on soil fertility including the growth behavior of bacteria (Kumar et al., 2013; Laldinthar and Dkhar, 2015; Wiesmeier et al., 2019; Garnaik et al., 2020). Bacterial isolates obtained were screened under different water potentials and also their plant growth-promoting traits were evaluated *in vitro*. Out of 167 rhizobacterial isolates, only five isolates survived at −0.73 MPa and were considered desiccation-tolerant. These bacterial strains were further characterized molecularly as *Enterobacter cloacae* BHUAS1, *Bacillus megaterium* BHUIESDAS3, *Bacillus megaterium* BHUIESDAS4, *Bacillus megaterium* BHUIESDAS5, and *Bacillus cereus* BHUAS2. These bacterial strains produced several enzymes such as amylase, catalase, cellulase, citrate, protease, and urease, which have been reported to have soil and plant beneficial effects (Bennett and Wallsgrove, 1994; Jaiswal et al., 2019).

The bacterial isolates selected also produced IAA, siderophore, and ACC deaminase enzyme, and they also solubilized insoluble phosphate under the impacts of desiccation stress. Studies reported that such beneficial PGP traits of bacteria positively influence plant growth and development during stressed conditions by increasing nutrient accessibility and protection against oxidative damage (Glick et al., 2007; Saikia et al., 2018). Studies have shown a positive impact of bacteria synthesized IAA on the morphology of plant roots as well as seed germination and growth of seedlings under the condition of osmotic stress, and they also help in the bio-fortification of micronutrients such as Zn, Se, Fe, and others (Ngumbi and Kloepper, 2016; Saikia et al., 2018; Khanna et al., 2022). Furthermore, phosphate solubilizing rhizobacteria isolated from drought-impacted agroecosystems was observed to be more beneficial for the improvement of plant health under the availability of limited water conditions (Sarma and Saikia, 2014). All five isolates tested here were efficient phosphate solubilizers both in control as well as under water stress. The isolates were also an efficient producer of siderophore under desiccation stress. Siderophore plays an essential role in maintaining the bioavailability of iron near the root zone (Sorty et al., 2016). Such siderophore producing bacteria have also been reported as a defensive agent for plants against heavy metal injury (Burd et al., 2000). The enzyme ACC deaminase producing rhizobacteria helps plants to tolerate stress by decreasing ethylene production through its deamination activity, which dissociates ACC into α-ketobutyrate and ammonia, thus inhibiting the production of stress-induced ethylene and thereby another downstream signaling (Glick, 2014; Gupta and Pandey, 2019). All five desiccation-tolerant rhizobacterial isolates tested here exhibited excellent ACC deaminase activity. The desiccation-tolerant rhizobacteria having ACC deaminase activity have been observed to trigger plant growth by increasing seed germination and pigment content under no stress (control) and desiccation stress by decreasing the level of ethylene along with increased production of ROS scavenging enzymes (Barnawal et al., 2012; Egamberdieva et al., 2019; Mukhtar et al., 2020). All five desiccation-tolerant bacterial strains exhibited significant activity of various antioxidant enzymes under no stress (control) and desiccation stress condition (Figure 3D). Thus, ACC deaminase activity along with other PGP properties has been observed to put an additive impact on plant growth and development under limited water condition (Saikia et al., 2018).

The desiccation-tolerant bacterial isolates secreted high amounts of EPS at −0.73 MPa water potential showing that limited availability of water induced the EPS production in them. The EPS produced by root adhered bacteria enhances the permeability of roots in soil by making soil aggregates and also maintains a positive water potential around the root zone, which results in improved water uptake and growth of the plant (Roberson and Firestone, 1992; Ali et al., 2014). These EPS produced by bacteria also protect seedling growth during water stress (Miller and Wood, 1996; Alami et al., 2000; Sandhya et al., 2009).

The present study also showed a protective role of desiccation-tolerant bacterial isolates in coping with the negative effects of water stress. Plant growth parameters got reduced under low water availability in non-treated plants. However, a significant increment in plant growth features was observed (Figure 4) in bacterial inoculated plants. In a study using pepper and tomato plants, inoculating the roots with PGPR was found to have improved biomass in comparison to un-inoculated plants under water stress conditions (Mayak et al., 2004; Kalozoumis et al., 2021). The isolate *E. cloaca*e BHUAS1 was one of the most effective strains with higher EPS production showing significant improvement in fresh and dry weight of wheat seedlings under water limiting conditions in comparison to untreated control plants. Similar results have been reported where EPS producing bacteria was significantly effective in reducing the negative effect of water stress on fresh weight, dry weight, and root and shoot length of the maize plant (Naseem and Bano, 2014; Vurukonda et al., 2016).

A major consequence of water stress was observed as a reduction in the chlorophyll content of wheat plants (Figure 5). During water stress, chloroplast gets damaged, which results in photoinhibition that reduces the ATP intake in the C_3_ cycle due to the low rate of electron transportation (Izanloo et al., 2008; Batool et al., 2020). The chlorophyll content should be enough to improve photosynthesis or stomatal conductance under water limiting conditions since two-thirds of the crop’s green part is actively involved in accumulating the light to drive photosynthesis (Ji et al., 2010; Batool et al., 2020). Therefore, the rate of photosynthesis is directly proportional to the amount of photosynthetic pigment (chlorophyll) per unit leaf area. Furthermore, the plant’s water status in form of leaf RWC and MSI treated with plant beneficial rhizobacteria are considered essential to keep up the stomatal conductance for CO_2_ exchange and execution of photosynthesis as well as proper operation of the electron transport system (Batool et al., 2020).

The amount of ROS and MDA accumulated in wheat plants under water stress conditions and in treatments with desiccation-tolerant bacterial strains showed a significant ameliorating impact on bacterial isolates (Figure 7). The amount of oxidative stress determines the intercellular concentration of MDA (Lulsdorf et al., 2013). The accumulation of MDA activates the cascade of signals characterized by various physiological aberrations in a cell such as a cell membrane becoming more permeable, decreased chlorophyll content, breakdown of macromolecules such as nucleic acids and proteins causing nutrients to remobilize extensively and premature senescence, and finally decreased crop growth and development. The MDA and ROS contents were observed to increase in un-inoculated plants during water stress (Figure 7), which is implicated in the reduced amount and capacity of antioxidants in wheat plants. It is well-reported that photosynthetic inhibition leads to the accumulation of ROS in cells and its organelles (Lulsdorf et al., 2013). Hence, it becomes mandatory to control the synthesis and accumulation of ROS within plant cells in order to check the damage caused by them in plants (Saneoka et al., 2004; Mahajan and Tuteja, 2005). Wheat plants having bacterial treatments showed lower MDA and ROS contents as compared to plants without bacteria showing an increased ROS scavenging ability under water stress.

The bacterial treated plants also exhibited higher activities of enzymes APX, CAT, and POD, and lower MDA and ROS contents (Figure 8). The results depicted that the synthesis of a suitable ROS scavenging antioxidant system in water-stressed plants under bacterial treatments might have boosted photosynthesis in treated plants. Similar results had been reported by Banik et al. (2016) in which it was concluded that bacterial treatment in *Agrostis palustris* showed drought tolerance by means of inhibiting cell membrane damage, decreased MDA production, and increased osmotic regulation.

Plants are reported to have the capability to regulate water relations in order to maintain cellular integrity during water stress conditions through osmotic adjustment by producing compatible solutes such as various soluble sugars, phenol, proline, and soluble proteins (Saharan and Nehra, 2011; Banik et al., 2016). These compatible solutes help plants to maintain cell turgidity and volume of the cell when water potential crucial for maintaining cell metabolism declines (Saneoka et al., 2004; Fischer, 2008; Batool et al., 2020). In addition, the osmolytes also assist in the repossession of metabolic activities of cells after overcoming stress (Abid et al., 2016).

Overall water unavailability was observed to impose a serious influence on wheat stress plant growth, yield, and physiological as well as biochemical functions. However, bacterial treatments helped the wheat plant to sustain its development and other functions proficiently, under the impacts of low water-induced drought stress. Wheat plants having bacterial treatments maintained higher biomass and root and shoot length. The plants with bacterial inoculation showed better MSI, RWC, chlorophyll, and carotenoid. These plants further displayed lower MDA and ROS and higher enzymatic activity of APX, CAT, and POD along with elevated production of soluble sugars, phenol, proline, and soluble protein. These isolates characterized may be used as potential candidates for the improvement in growth and yield of wheat under conditions of low water availability or any other similar stress.

## Data availability statement

The original contributions presented in this study are included in this article/supplementary material, further inquiries can be directed to the corresponding author.

## Author contributions

AS collected the samples, executed the experiments and data analysis, comprehended the study, arranged the figures, and wrote the first draft. VP edited the manuscript. Both authors conceived the study and approved the final manuscript.
